# A Framework for Competencies for the Use of Mobile Technologies in Psychiatry and Medicine: Scoping Review

**DOI:** 10.2196/12229

**Published:** 2020-02-21

**Authors:** Donald Hilty, Steven Chan, John Torous, John Luo, Robert Boland

**Affiliations:** 1 VA Northern California Health Care System Mental Health & Department of Psychiatry and Behavioral Sciences UC Davis School of Medicine Mather, CA United States; 2 Palo Alto VA Health Care System Palo Alto, CA United States; 3 Beth Israel Deaconess Medical Center Psychiatry Harvard School of Medicine Boston, MA United States; 4 Consultation-Liaison & Emergency Psychiatry UC Irvine Department of Psychiatry UCI Health Irvine, CA United States; 5 Harvard Longwood Psychiatry Residency Training Program Brigham and Women's/Faulkner Hospitals Harvard Medical School Boston, MA United States

**Keywords:** apps, behavior, education, mobile, outcome, competency, technology, health, mobile phone, framework

## Abstract

**Background:**

To ensure quality care, clinicians need skills, knowledge, and attitudes related to technology that can be measured.

**Objective:**

This paper sought out competencies for mobile technologies and/or an approach to define them.

**Methods:**

A scoping review was conducted to answer the following research question, “What skills are needed for clinicians and trainees to provide quality care via mHealth, have they been published, and how can they be made measurable and reproducible to teach and assess them?” The review was conducted in accordance with the 6-stage scoping review process starting with a keyword search in PubMed/Medical Literature Analysis and Retrieval System Online, APA PsycNET, Cochrane, EMBASE, PsycINFO, Web of Science, and Scopus. The literature search focused on keywords in 4 concept areas: (1) competencies, (2) mobile technologies, (3) telemedicine mode, and (4) health. Moreover, 2 authors independently, in parallel, screened the search results for potentially relevant studies based on titles and abstracts. The authors reviewed the full-text articles for final inclusion based on inclusion/exclusion criteria. Inclusion criteria were keywords used from concept area 1 (competencies) and 2 (mobile technologies) and either 3 (telemedicine mode) or 4 (health). Exclusion criteria included, but were not limited to, keywords used from a concept area in isolation, discussion of skills abstractly, outline or listing of what clinicians need without detail, and listing immeasurable behaviors.

**Results:**

From a total of 1232 results, the authors found 78 papers eligible for a full-text review and found 14 papers directly relevant to the 4 key concepts. Although few studies specifically discussed skills, the majority were clinical studies, and the literature included no lists of measurable behaviors or competency sets for mobile technology. Therefore, a framework for mobile technology competencies was built according to the review, expert consensus, and recommendations of the Institute of Medicine’s Health Professions Education Summit and Accreditation Council of Graduate Medical Education framework. This framework borrows from existing competency framework domains in telepsychiatry and social media (patient care, medical knowledge, practice-based learning and improvement, systems-based practice, professionalism, and interpersonal skills and communication) and added domains of mHealth clinical decision support, device/technology assessment/selection, and information flow management across an electronic health record platform. mHealth Asynchronous components require additional traditional learning, teaching, supervisory and evaluation practices. Interactive curricula with case-, problem-, and system-based teaching may help faculty focus on decision making and shape skills and attitudes to complement clinical exposure.

**Conclusions:**

Research is needed on how to customize implementation and evaluation of mHealth competencies and to ensure skill development is linked to the quality of care. This will require the management of organizational change with technology and the creation of a positive electronic culture in a complex policy and regulatory environment.

## Introduction

### Background

Mobile technologies such as mobile phones and other devices are supported by third generation and fourth generation mobile networks for data transport, computing, and integration. They have been a force in business, entertainment, and health communities and enable communication, monitoring, consulting, and other health care services across geographical, cost, and temporal barriers [1]. This movement is consistent with person- and patient-centered care, often spoken of as participatory medicine. It has moved patients from being mere passengers to responsible drivers of their health, and physicians value them as partners [2]. Accordingly, educational reform with technology is suggested by the World Health Organization [3] and the Institute of Medicine [4] to help physicians learn about technologies and educate patients.

**Figure 1:**
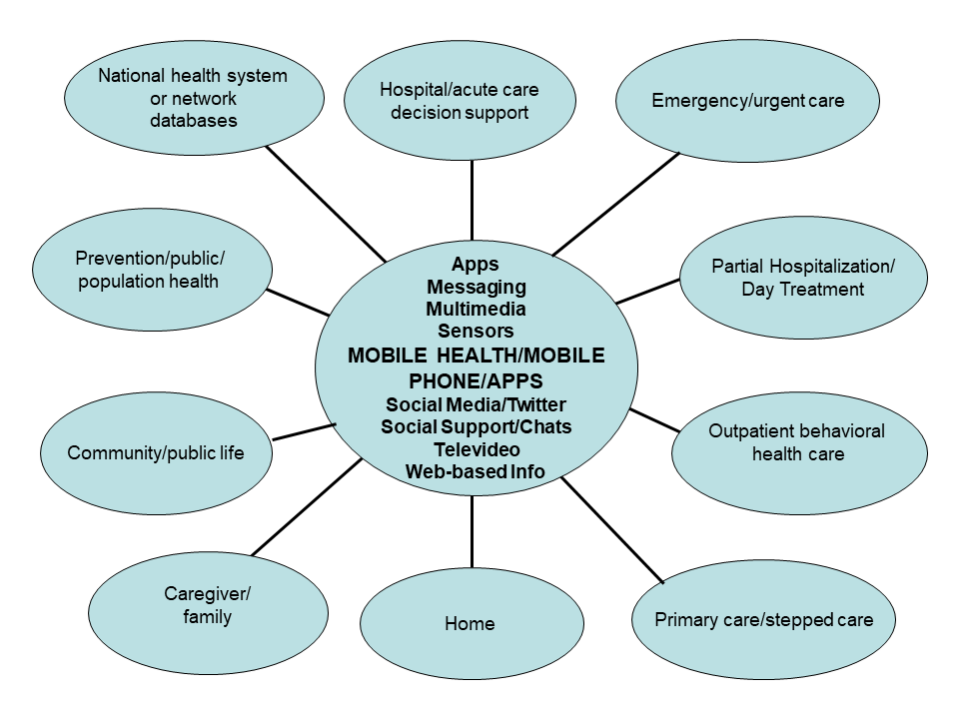
How mobile health, mobile phone/device, and apps integrate information in the digital age.

In health care, mobile health (mHealth) components include monitoring, alerting, data collection, record maintenance, and detection and prevention systems [5]. mHealth was previously defined as “unwired e-med,” [6] then as mobile communications and network technologies [7], and now as the application of mobile or wireless communication technologies to health and health care [8]. mHealth service architecture includes many settings, devices, and operational features (Figure 1). These afford accessibility, timeliness, and integration. Technology enables providers to do more with patients in a longitudinal, integrated way and is therefore called a *practice extender* [9].

mHealth recontextualizes health care communication via phones, tablet computers, and wearable devices (eg, smart watches and sensors) [10,11]. As such, mHealth intersects with the field of remote patient monitoring of patients outside of conventional clinical settings (eg, home-based chronic disease management). Persons, patients, caregivers, and family members report more support if a problem arises and have fewer emergency department visits and hospitalizations [10,11]. Mobile apps offer (1) portability for access to data, systems, and other information, regardless of patient geography and transportation barriers; (2) an inexpensive option vs traditional desktop computers; and (3) additional features such as context-aware interventions and sensors [12] with real-time feedback. Although mobile technologies may feature live streaming of data, they are typically used like the 24-hour, 7-day per week Holter monitor in cardiology, which is read intermittently at the end of the data collection; therefore, it is usually functionally asynchronous.

The mHealth devices (Figure 1) have the following features [13]:

- Voice/video calling: convenient way for clinicians and patients to remotely communicate;
- SMS and multimedia message services: transmit text messages and video clips/sound files as a cost-effective way to deliver education;
- Multimedia functions: provide a range of learning opportunities;
- Inbuilt sensors: touch, motion, and GPS sensors that simplify clinical assessment and lifestyle and social activities;
- Device connectivity: practical and less error-prone data entry than manual processes.

mHealth also has clinical decision support (CDS) and information flow management features, which helps providers, patients, and others make decisions *in time*. These features improve outcomes, reduce unnecessary mistakes, and increase efficiency [14-16]. Health care has different types of information systems and domains, including the electronic health record (EHR), picture archiving and communication systems, laboratory information systems, and CDS systems. CDS provides clinicians, patients, and others with knowledge and person-specific information, intelligently filtered or presented at appropriate times, to enhance health and health care. Research is investigating core components and processing features [13], including in child and adolescent psychiatry [15]. CDS arose within clinical informatics but is increasingly valued in medicine and behavioral health [16].

### Competencies for Technology

Clinicians need a framework and skills/competencies for mHealth as a way to link skill and attitudinal change with quality of care [17]. The Institute of Medicine’s core competencies for the health professions—now being applied to telepsychiatry and other technologies—include the ability to provide patient-centered care, work in interdisciplinary teams, employ evidence-based practice, apply quality improvement (QI), *and* use information technology [4]. Competency-based medical education movement focuses on clinical skill development and curricula to produce desired outcomes for learners in addition to knowledge acquisition [18]. Learner-centered educational outcomes are set, and then teaching and assessment methods are aligned [18,19]. Faculty assess learners during patient care in addition to seminars to ensure skill development [20,21].

A straightforward competency framework for some technologies is available for faculty, program directors, and administrators (eg, telepsychiatry and social media) [22-24]. It is based on the US Accreditation Council of Graduate Medical Education (ACGME) framework [25], which has domains related to patient care, medical knowledge, practice-based learning and improvement, systems-based practice, professionalism, and interpersonal skills and communication [25]. Another useful framework is the Royal College’s Canadian Medical Education Directives for Specialists, which uses 7 roles that all physicians play: medical expert, communicator, collaborator, manager, health advocate, scholar, and professional [26].

The telepsychiatry competencies framework simplified the Dreyfus 5-level model of learners (level 1–novice; level 2–advanced; level 3–competent; level 4–proficient; and level 5–expert) [27] to 3 levels: novice/advanced beginner (eg, early clinicians or those unfamiliar with technology); competent/proficient (eg, able to translate in-person to technology-based care well); and expert (eg, advanced in clinical care and via technology). Others use a similar gradation nationally, such as the National Hospice and Palliative Care Organization [28].

The telepsychiatry *patient care* domain was divided into 2 parts: (1) clinical—the history, interviewing, assessment, and treatment and (2) administrative-based procedures/issues related to care such as documentation, EHR, medicolegal aspects, billing, and privacy/confidentiality. *Systems-based practice* included interprofessional education models of care and safety, whereas *professionalism* included integrity, ethics, culture, and diversity. As both telepsychiatry and in-person care are synchronous practices, the competencies are similar to a few significant and many minor adjustments in approach, execution, and evaluation (eg, inquiry about use of and comfort with technology and modification of a mental status examination at a distance). The competencies also added other important features such as detailed andragogy/pedagogy methods for teaching and assessment of learners, faculty development priorities, and institutional competencies for administration [22].

Social media and networking competencies apply as a preview of mHealth’s asynchronous components [23]. Social media (1) is asynchronous not synchronous, so it cannot be *organized* or structured like traditional care; (2) may affect how participants engage within the therapeutic frame; (3) is conducted over public, private, and health system sites, making data integration and security difficult, if not impossible; (4) challenges users to maintain tight personal and professional boundaries, as email and texting may cause complications; and (5) requires clinicians to verify the identity of the patient for a social media account, as false identities are sometimes used [24]. A history about the use of social media needs to inquire about social media sites visited as well as for what purpose they are used [29].

For *patient care* related to social media, the competent/proficient clinician discusses technology during the consent process and screens for social media use. The clinician decides with the patient whether social media is *part of* the clinical service contract. This requires some reflection on its pros/cons, as much of it is outside the therapeutic hour [10]. At a minimum, if it is part of the plan, it should be used as part of an established doctor-patient relationship. The clinician needs to systematically screen what is used and for what purpose(s) (eg, entertainment, health care, and behavioral health care). A plan may be needed to manage risks (eg, privacy, self-disclosure, and cyberbullying). As clinicians also have social media profiles, they have to be mindful of colleagues and patients–to portray a professional image–and to remember that one represents oneself, the institution, and the profession. Many of these challenges apply to mHealth, so additional screening and planning are needed with regard to patient care [17].

### Lack of Existing Competencies for Mobile Health

mHealth is used clinician-to-clinician, clinician-to-patient, and person/people-to-others; the participants may be mobile or stationary. This poses significant challenges to clinical care, as mHealth alters communication, boundaries, and privacy/confidentiality; therefore, clinicians are encouraged to screen what technology is being used, how, and when [30]. mHealth may therefore affect the therapeutic relationship and it is important to use the *right* technology at the *right* time (eg, not using an app or text to express suicidal ideation [SI]) as part of a treatment plan. Although mHealth may empower patients via in-time learning and increased self-efficacy, all parties need to have time to acquire knowledge, gain skills, and adjust attitudes. This is greater than any single party seeking information, as knowledge does not necessarily translate into skill.

A conceptual approach may need to consider mHealth as both *inside* and *outside* of the clinical visit. Patients bring up apps, communications, and assignments from clinicians (eg, filling out a questionnaire). This is a new dimension of care typically without problems. An approach on competencies, however, encourages the clinician to use mHealth in the treatment plan more purposely (eg, using an app weekly for monitoring depression), rather than spontaneously. The clinician can help the patient use an app in a (structured) way that feeds into the EHR—instead of a half dozen apps that do not—which simplifies treatment and protects privacy. mHealth may also be used for communication outside the visit, and if a clinician uses her/his personal device for professional care, this may be disruptive, as texts and email create extra workload and irregular contact after hours (ie, a boundary problem).

There are things that mHealth, telemedicine/telepsychiatry (ie, video), social media, and other technologies have in common. mHealth like video connects participants synchronously (eg, live feed of data to a clinician for decisions) [31] or asynchronously [32]. Additional competencies are needed as mHealth includes CDS, mobile technology assessment/selection, and information flow management across an EHR platform. Unlike telepsychiatry, but like social media, mHealth may have asynchronous components (eg, texting) [33]. Not all patients may be suitable for mHealth and social media because of impulsivity (eg, disclosure of information, attempts at after-hours contact), and otherwise failing to understand the medium. On the other hand, some severely ill patients may benefit from mobile technologies. They may provide a *wraparound* approach, similar to case managers, for patients with schizophrenia who live in the community.

In addition to the clinical care adjustments, there are educational ramifications for mHealth competencies. The examples below show how supervision is on one hand similar (eg, mobile technologies are discussed like any other topic at a weekly supervisory meeting) but on the other hand, different (eg, additional supervisory contact to review online data about patient experiences over time). Accordingly, leaders need to organize a curriculum, perform program evaluation, train faculty, and administer change.

This paper will help with reference to mobile technologies’ (ie, mHealth, mobile phones and other devices, and apps) competencies so the reader can:

1. develop competencies for mobile technologies using the ACGME framework, which is founded in the competency-based medical education movement,
2. model the mHealth competencies based on the competencies for telepsychiatry and social media, but shift them based on mHealth’s components, concepts, operations, and processes,
3. model teaching and evaluation processes for clinicians, programs directors, and health care systems, which facilitate skill development based on those for telepsychiatry and social media but adjust them contextually based on mHealth’s components, concepts, operations, and processes.

**Figure 2:**
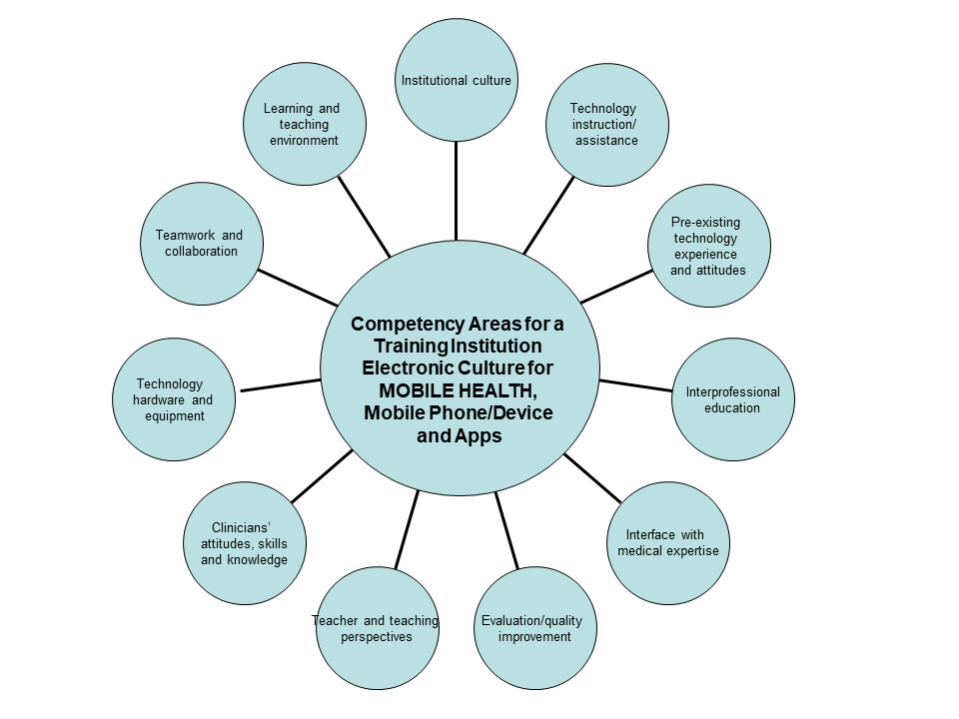
Competency areas of an e-Culture for a training institution related to mobile health, mobile phone/device, and apps.

**Figure 3:**
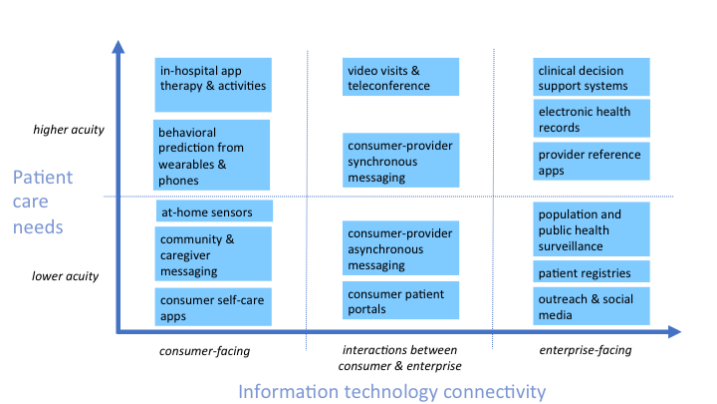
The relationship between patient care needs based on acuity and information technology connectivity.

The section *Methods* outline the approach, strategy, validity assessment, and expert opinion processes. The *Results* are first organized into the findings, how the framework was built, and an overview of the competency movement in medicine. Existing competencies in telepsychiatry and social media are briefly described to provide historical background. Next, the *Results* outline unique elements of mHealth, with Figure 1 to provide clinical context, and a competency set, with examples to provide specifics. Some examples focus on clinical and supervisory themes, whereas others focus on teaching by faculty, with tables to outline both learning (Multimedia Appendix 1) and teaching (Multimedia Appendix 2) specifics. Figure 2 provides an overall picture on components of an e-learning culture and Figure 3 shows how the patient, clinician, and system need an interface.

## Methods

### Approach

The literature keyword search was conducted from July 2003 to February 2019. The philosophical approach to the search was used according to the original 6-stage process [34] and updated modifications [35] for scoping reviews. These reviews are typically undertaken to examine the extent, range, and nature of research in a topic area and identify gaps in knowledge rather than examine more specific, narrow topics based on study designs of systematic reviews. Both types of reviews use an approach based on concept, target population, and health outcomes.

The stages in this process have been described as (1) linking a clear purpose with a well-defined research question, with a rationale for completion; (2) identifying relevant studies based on the question and purpose, employing a suitable team; (3) selecting studies based on an iterative process involving searching the literature, refining the search strategy, and reviewing articles for study inclusion, along with reviewer discussion at the beginning, midpoint, and final stages; (4) charting the data and updating the form by having at least 2 reviewers extract information; (5) analysis, reporting, and considering the meaning of the findings (previously known as collating, summarizing, and reporting); and (6) using preliminary findings to obtain consultation from stakeholders toward an aim, using a plan for how data are collected, analyzed, reported, and integrated within the overall study outcome. Finally, clarifying terminology (eg, scoping reviews vs studies) and quality assessment are suggested [35].

### The Research Question

The question that guided the review was, “What skills are needed for clinicians and trainees to provide quality care via mHealth, have they been published, and how can they be made measurable and reproducible to teach and assess them?” The goal was to identify behaviors (skills and competencies), make them measurable for implementation, and be able to assess learning outcomes, which are distinct from clinical treatment and service system outcomes. Implementation involves assessment of acceptability, adoption, appropriateness, feasibility, fidelity, implementation cost, penetration, and sustainability [36]. As the search proceeded, additional terms were suggested by experts to potentially modify of the question, but new searches did not result in additional data, and the question did not change.

### Identifying Relevant Studies: The Search Strategy

A literature keyword search from July 2003 to February 2019 (previously described) [30] placed keywords into concept areas [37]. The databases searched were PubMed/Medical Literature Analysis and Retrieval System Online, APA PsycNET, Cochrane, EMBASE, PsycINFO, Web of Science and Scopus, Science Citation Index, Social Sciences Citation Index, Telemedicine Information Exchange database, Centre for Reviews and Dissemination, and The Cochrane Library Controlled Trial Registry database. The initial literature search targeted 4 concept areas: (1) competency(ies) (skills, behavior, cognition, cognitive, pedagogy, framework, education, training, milestones, and curriculum); (2) mobile technologies (Web-based, apps, text, internet, mobile phone, wearable, and device); (3) telemedicine mode (video, synchronous, asynchronous, phone, and email); and (4) health (patient, clinician, care, services, medicine, psychiatry, mental, and behavioral).

### Study Selection

An iterative process involving searching the literature, refining the search strategy, and reviewing articles for study inclusion was used. Two authors (DH and SC) independently, in parallel, screened the search results for potentially relevant studies based on titles and abstracts. Full-text articles were reviewed for final inclusion based on the keyword search. Inclusion criteria were keywords used from concept area 1 (competencies) and 2 (mobile technologies) and either 3 (telemedicine mode) or 4 (health). Exclusion criteria included keywords used from a concept area in isolation (eg, using the word competency but not mentioning a single skill); discussing skills abstractly (eg, as part of clinical skill development), outline or listing of what clinicians need (eg, knowledge, skills, and attitudes) without detail, listing behaviors that are not measurable (eg, good engagement), and terms in combination (eg, cognitive, milestones, and patient) without discussing competencies).

Findings of the searches were shared with others at the beginning of the process to decide study inclusion and exclusion, for the 2 reviewers to independently review abstracts and full papers; when disagreements on study inclusion occurred, a third reviewer determined the outcome. Reviewers met at the beginning, midpoint, and final stages of the abstract review process to discuss challenges and uncertainties related to study selection and to go back and to refine the search strategy. Study selection would have involved *posthoc* inclusion and exclusion criteria, based on the specifics of the research question, new familiarity with the subject matter, and expert input. However, none were added in this study.

### Charting the Data

A data-charting form was not developed and used to extract data from each study, but notes were organized consistent with a narrative review or descriptive analytical methods by each reviewer to extract contextual or process-oriented information from each study, particularly the frameworks of telepsychiatric and social media competencies. The reviewers then compared and consolidated information regarding content. A qualitative content analysis approach would have been used if there was more content, to make sense of the wealth of extracted data. A descriptive analytical method was used to summarize the process and content information of discussions with experts, in an effort to chart and summarize complex concepts in a meaningful way.

### Analysis, Reporting, and Considering the Meaning of the Findings

This phase was to organize meaningful results in a table, study by study, with skills outlined and parsed together incrementally. Then, the authors consolidated the data and followed up with the expert consensus step. There were few papers, so the findings were reported individually, as the depth of existing research was less than expected. Indeed, there were virtually no experts outside of the authors with a command over the necessary different fields (eg, pedagogy, mHealth, and medical education administration). Therefore, the reporting of results and applying of meaning to the results was added to the authors’ preliminary framework tables, and case examples were used to describe the findings. A descriptive numerical summary of results and a thematic analysis were not possible.

### Consultation for Expert Opinion

Expert opinion was solicited in 4 ways: (1) a series of medical educator conference calls focused on teaching competencies [22,23], (2) discussion during several regional and national presentations (eg, American Association for Directors of Psychiatry Residency Training, (3) through individual discussions with educational experts [23], and (4) input from national behavioral health organizations–for example, psychiatry/medicine, psychology, social work, counseling, marriage/family, psychiatric nursing, and behavioral analysis) via 2 rounds of input for the consensus process [22,38]. That process was based on an already published review of interprofessional literature (ie, psychiatry/medicine, psychology, social work, counseling, marriage/family, psychiatric nursing, and behavioral analysis), which gained 2 rounds of input from national organizations as part of the consensus process [38].

The participants included educational leaders (eg, course/program directors, chairs, deans, a national society executive director), educational researchers, journal editors, and authors of educational textbooks. Participants had content expertise in medicine, psychiatry, education, health services, mobile technologies, and ethics. They represented viewpoints enriched by their leadership roles within their professional societies. Stakeholders were consulted with a purpose to validate preliminary findings, to integrate additional data related to the findings, and to revise the search to collect better data, if possible. Using a modified Delphi process, the conference participants reviewed an initial framework [22] based on qualitative analysis of identified themes that incorporated both ACGME competencies and the Royal College’s Canadian Medical Education Directives for Specialists roles. The Delphi process is based on the principle that decisions from a structured group of individuals are more accurate than those from unstructured groups.

The conference calls series comprised 2 groups of 8 medical educators from the United States and Canada to discuss educational competency development. The preliminary findings were placed in the framework table, with both themes and individual suggestions (ie, findings). This allowed stakeholders to build on the evidence and offer a higher level of meaning, content expertise, and perspective to the preliminary findings. The references were also reviewed, and additional references were solicited. The Delphi process was modified in 3 ways: conferences were conducted by video/telephone, they occurred using groups of people from more than one organization, and part of the group feedback was provided by returning questionnaires.

## Results

### Overview

From a total of 1232 potential references, the authors found 78 eligible for a full-text review and 14 papers directly relevant to the concepts. From papers’ references, another 10 papers were found, but they were mainly foundational sources about competencies from continuing and graduate education. There were a few papers on skill development, mainly in nurses and community health workers [39-41]. There were many references to patient education and the quality of a good app. There were also many references to informatics competencies, but none with competencies for mHealth; however, one used an ACGME framework [42].

Therefore, a framework was built according to the review, expert consensus, and recommendations of the Institute of Medicine’s Health Professions Educational Summit [4] and ACGME framework [25]. It borrowed from existing frameworks in telepsychiatry, social media, and telebehavioral health. As mobile technologies have similarities to in-person and telepsychiatric care, mHealth competencies were placed in milestone domains of patient care, medical knowledge, practice-based learning and improvement, systems-based practice, professionalism, and interpersonal skills and communication.

Additional competencies were suggested as mHealth includes CDS, device/technology assessment/selection, and information flow management across an EHR platform. As care with mHealth may have asynchronous components—such as social media—competencies for trainees and clinicians may help them shift traditional learning, teaching, supervisory, and evaluation practices to achieve targeted outcomes. Asynchronous is defined in several ways but it includes sending information byte by byte, sequentially between parties (eg, texting), radiographs/pictures (eg, radiology and dermatology), and prerecorded information transfer [32].

### Mobile Technologies Competencies

The experts agreed that the mHealth competency set be modeled after the ACGME framework of the telepsychiatric and social media competencies [22-24] and employ 3 levels named novice/beginner, competent/proficient, and expert [22,28]. The framework has been described in Multimedia Appendix 1.

### Example 1: Description of Patient Care History Taking, Engagement, Assessment, and Treatment

The Patient Care section includes history taking, engagement and interpersonal skills, assessment, education and management, and treatment planning. It also includes administration, documentation, and medicolegal issues such as privacy, confidentiality, safety, data protection/integrity, and security. Clinicians should help patients reflect on the pros/cons of mobile technologies’ use as part of ongoing treatment and document this (eg, as part of the consent form or in a progress note). This may include, but not be limited to, the competent/proficient clinician selecting the mobile technology option based on patient preference, skill and need (ie, purpose). To do that, it is helpful to know if the patient uses mobile technologies for personal life, health care, and behavioral health care. The clinician should see if the patient is aware of risks (eg, privacy, self-disclosure, and potential for cyberbullying) and help them select options that are easy to use.

Technology in the form of mobile technologies can be useful for preparing for a treatment session or collecting information between sessions (eg, see the row titled “Management and treatment planning” in Multimedia Appendix 1). Ecological momentary assessment (EMA) involves repeated sampling of naturalistic behaviors and experiences [43-45] of day-to-day life such as habits (eg, smoking), mood changes (ie, depression), physical activity, and vital signs (eg, blood pressure). Paper-and-pencil diary methods (eg, medication calendars) are subject to memory lapses, recall bias, and bias related to social desirability. Now, mobile technologies may immediately capture information by alarms (ie, signal dependent) or key events (ie, event-dependent) that facilitate in-person trajectories and temporal sequences of behavior, particularly if wearable sensors are used.

In behavioral health, EMA measurement of changes in mood/affect correlate better with clinician-rated affective symptoms, may be used to detect subsequent risk of SI in bipolar patients [46], and may be preferable to patients (eg, Veterans prefer to complete psychometric measures such as the Patient Health Questionnaire or PHQ-9 using an iPhone [47,48]). If an urgent issue arises (eg, a patient reports SI via an app), an immediate telephone call or emergency response is almost always suggested, although a personalized email or text may be therapeutic and successful to prevent worsening [49-52].

### Teaching, Assessment, and Evaluation of Mobile Technologies

#### Overview

The outcome (ie, competency skill or behavior) should predetermine its measurement as well as teaching, supervision, and organization of clinical services. This is particularly important as mobile technologies cause a shift, which may include events as part of a regular clinical visit or between visits. If clinicians who are supervisors pre-emptively decide that mobile technologies are not part of care or informally approach mHealth, trainees may not provide adequate supervision to develop necessary skills. Similarly, poor outcomes may occur if the discussion of mobile technologies is left to chance rather than a planned part of supervision.

The supervisor’s approach requires many things, particularly a solid foundation in psychiatry (eg, the therapeutic frame and boundary issues) and experience with technology. She/he needs clear personal and professional boundaries and professional-personal wellness/balance to avoid other problems. She/he must plan how to monitor information flow and make decisions, if applicable, between visits. Patients and clinicians should have an initial discussion and monitor changes. Attention to longitudinal documentation is needed (eg, consent form and progress notes) for both supervisors and trainees. A patient’s increasing number of requests for nonphone contact between visits (eg, apps, texts, and emails) may be a good sign or signal expectations that are not healthy.

### Clinical Supervision of Mobile Technologies Competencies

An approach to teaching these competencies involves a wide range of methodologies, settings, and participants (Multimedia Appendix 2). Mobile technologies and social media have asynchronous functions, so an approach to *organize* the teaching plan is needed. A computer can be programmed to email, text, or otherwise contact a patient or a clinician may contact the patient throughout the week or vice versa. The flow of information has to be funneled *into* scheduled supervision as part of a caseload or quickly dealt with by a *curbside consultation* *in time*. Thus, clinical workflow and administrative policies may be required to provide a trainee time to reflect, consider options, and get advice before responding.

### Traditional Teaching Approaches

*Case-based learning* (as seen in Multimedia Appendix 2) is a good teaching and learning method that uses real life examples or vignettes in seminars, site-based case conferences, and QI/grand round presentations. These also draw from trainees’ experiences with patients about mobile technologies. Interactive methods such as role-plays can be used to flush out the issues, practice communication skills, identify options for decisions, and propose solutions for patients. Context for other settings and in-depth learning occurs through group input and feedback from peers and faculty. Furthermore, this provides an opportunity to build and solidify the resident *Role as an Educator* (Multimedia Appendix 2). She or he learns to work with an interprofessional team and adapt communication skills to multiple people. For a content area such as *Knowledge* (Multimedia Appendix 1), decision support tools may help clinicians evaluate apps to see if they are evidence based and develop an approach to use them in an evidence-based fashion.

Faculty cannot supervise mHealth care in real time like a scheduled visit. Reflection, peer advice, and faculty supervision may be required quickly, which may necessitate on-site *on-call* or *faculty of the day* supervision, but these other faculty may triage a situation differently than the trainee’s ongoing supervisor. With regard to Example 1 given above related to SI, the trainee has to decide what to do and has several potential options: do nothing (if it is a chronic behavior for the patient); send a personalized, empathic text that is therapeutic [49]; telephone the patient; and trigger an emergency response. The personalized text may be part of an ongoing therapy for nonlethal impulsive harm (eg, self-mutilation). All parties should learn about clinic, department, and health system policies—if any are in place—or be prompted to develop them.

### Example 2: Description of Supervision Clinical Care With Patients by Observing Faculty

CDS tools may also help with diagnosis and treatment [53] and this is learned in the flow of clinical care rather than by seminar, although case presentation and QI projects may help others learn and improve workflow. CDS is sometimes misunderstood as alert, notification, or explicit care suggestions, but CDS encompasses a variety of tools including, but not limited to computerized alerts and reminders for clinicians and patients, clinical guidelines, condition-specific order sets, focused patient data reports and summaries, documentation templates, diagnostic support, and contextually relevant reference information [54].

CDS tools provide clinicians, patients, and others with knowledge and person-specific information, intelligently filtered and presented in a timely fashion. These help to enhance health and health care by enhancing decision making in the clinical workflow.

Examples are patient-report questionnaires and rating scales, which standardize evaluation and facilitate treatment tracking by automatically sending scores to a clinician in real time. One option (eg, Outcomes Questionnaires Analyst) utilizes electronic tracking of distress among patients and it has been used by some health systems with other disease-specific scales in order to within an online behavioral health EHR to inform decision making. Another uses the Brief Symptom Inventory for monitoring [52]. A Web-based CDS system for depression care management helps care managers and others implement the collaborative care model [55].

## Discussion

### Overview

Mobile technologies have similarities and differences to in-person and telepsychiatric care. The competencies for mobile technologies for trainees and faculty are based on graduate medical education, but they apply across health disciplines, professions, and behavioral health. Program directors, faculty, department leaders, and health system administrators must help trainees make decisions on how to best assess, triage, and treat patients and maintain the therapeutic relationship while using technology. Clinicians/faculty can model the importance of placing the patient’s needs first and embracing technology for health care reform [3,4]. If and when they do not, students’ digital professionalism has been shown to deteriorate during core clinical clerkships, according to behavior, privacy, and attitudinal measures [56].

Traditionally, clinicians depend on research and clinical measures as well as guidelines for care. The Healthcare Information and Management Systems Society (HIMSS) has created assessment guidelines for mobile technologies [57], and existing evidence-based guidelines for apps stratify purposeful use, content/process, measurement/assessment, and quality [30,58-61]. However, skills are needed to use the evidence base, and unfortunately, some *guidelines* on email, social media, and other technologies are not evidence- and consensus-based [17,39].

### The Future State

Going forward with mHealth competencies, there are several suggestions. The findings need more detailed metrics and thorough evaluation to be measurable. For both cross-sectional and longitudinal trajectories, qualitative and quantitative evaluation of participants is suggested to iteratively improve the process. Research is also needed on to how to implement competencies in a customized way and evaluate them to ensure skill development improves quality of care. Organizational assessment and change are needed as this mHealth is a paradigm shift that recontextualizes digital health care. Trainees are a helpful *vehicle* for teaching faculty about mHealth, social media and other technologies—in clinical settings and particularly through QI, scholarship/research projects, and grants (eg, an Institute on Medicine as a profession and the Josiah Macy Jr Foundation 2-year grant on social media).

Technology significantly shapes people’s lives, and they have expectations in health care as in the rest of the real world. Undergraduate universities, business, banking, and even dating services learned that to prosper and survive, they had to adjust to people’s preferences for electronic and online modalities [62]. The business approach to new markets and to match products with user needs (Figure 3 [63]) could be useful for medicine related to technology. For patients’ health care to improve, clinicians need to understand the person behind the patient, their motivations, and their behaviors [64]. To do this, new paradigms are needed to help organizations change [31,65-68], and institutional competencies for technology have been suggested for academic health centers [22]. At a minimum, a plan for technology infrastructure and policy/procedures are needed for clinical, education, and research missions. For mobile technologies, there are at least 5 paradigm shifts at hand—each driven by demand, outcomes, competencies, and evaluation:

1. Patients *and* trainees of the X, Millennial/Y, and Z generations want *and* expect a digital health care experience [23,24];
2. Technology-based health care is *at least as efficacious* (eg, telepsychiatry) as in-person care, and it leverages resources much more efficiently [32,56,69];
3. Health care systems must focus on skills/competencies *in addition to* knowledge to ensure quality, safety, and efficiency of care;
4. An EHR-platform informed by information systems should be a versatile, flexible foundation for *good* clinical care (eg, multiple entry portals via mHealth) [64];
5. The mHealth is an example of a new and strategically better way to *frame* or organize health care [8,13,23] *if* leaders and other participants embrace it and find a way to manage constraints (eg, reimbursement).

### Shifts in Education and Practice

The path through training, lifelong practice, and accreditation has some disconnections despite common interests related to policy, regulatory, and other matters [30]. In behavioral health, coordination and collaboration may involve the Association of State and Provincial Psychology Board, the American Board of Psychiatry and Neurology, and the American Psychiatric Association. Legal and regulatory issues are complex as clinicians need to adhere to in-person and telehealth-relevant laws and requirements—and adapt those standards to mHealth—while attending to contextual and overarching jurisdictional issues of states and the government (and its agencies). Nongovernmental regulatory requirements and recommendations may also apply (eg, in the United States, Joint Commission, Council on Accreditation, Utilization Review Accreditation Commission, and HIMSS).

There are limitations to this set of mobile technologies competencies. First, study selection was based on guidance from a team (ie, only 2 reviewers), which was not interdisciplinary, and without a transparent and replicable process. Second, a data-charting form was not developed and used to extract data from each study. Third, breaking the analysis phase into meaningful and systematic steps would have been more rigorous and would have provided a guide for future researchers [70]. Fourth, with regard to reporting and considering the meaning of the findings, only a thematic analysis was presented, rather than a numerical analysis of the extent and nature of studies. A summary of results would be in order, but when a scoping review is done when there is *insufficient evidence*, that is not always possible [71]. Fifth, broader input for consensus across organizations (eg, American/British/Canadian Medical Associations and American Telemedicine Association) could have been helpful. Sixth, although *posthoc* changes via experts were added to the table of competencies, there were few because of the authors’ familiarity with the subject matter being far ahead of the literature and the experts obtained. A qualitative, small group interview approach with experts via a semistructured guide could have asked participants to identify models for care (regardless of whether they were published) and to specify key model components [71]. Finally, if calls had been recorded, summarized, and shared with the group, ideas could have been clarified and additional input gained.

## Appendix

Multimedia Appendix 1 A Framework to Adapt Accreditation Council of Graduate Medical Education (ACGME) Core Competencies to Mobile Technologies Clinical Competencies.

Multimedia Appendix 2 Teaching, Assessment, and Evaluation Methods for Mobile Technologies (Mobile Health, Mobile Phone, and Apps) Clinical Competencies.

## Abbreviations

ACGME: Accreditation Council of Graduate Medical Education
CDS: clinical decision support
EHR: electronic health record
EMA: ecological momentary assessment
HIMSS: Healthcare Information and Management Systems Society
mHealth: mobile health
OQ: Outcomes Questionnaire
QI: quality improvement
SI: suicidal ideation
