# Supplementary material for: A Framework for Competencies for the Use of Mobile Technologies in Psychiatry and Medicine: Scoping Review
Source: JMIR Mhealth Uhealth. 2020 Feb 21;8(2):e12229. doi: 10.2196/12229 (PMC7060500; doi:10.2196/12229)
Supplement: Multimedia Appendix 1 [file mhealth_v8i2e12229_app1.docx]

Multimedia Appendix 1. A Framework to Adapt Accreditation Council of Graduate Medical Education (ACGME) Core Competencies to Mobile Technologies Clinical Competencies.

| Area/topic^a^ | Novice/advanced beginner (ACGME milestone level 1-2) | Competent/proficient (ACGME milestone level 3-4) | Advanced/expert (ACGME milestone level 5) |
| --- | --- | --- | --- |
| **Patient care** | | | |
| History taking | Add questions such as:   - Are you using mobile technologies and for what?   - Fun/social?   - Health? - Would you like to use it/these for health care, if available? | Screen systematically with questions such as:   - Which mobile technologies do you use: exercise? entertainment? social? health? - For health care?   - Communicate with your medical doctor (MD), nurse (RN) or others?   - Discuss mental health issues? - Do you use mobile technologies more or less than other technologies (email/text, apps, internet, social media? - What are the pros/cons?   - Are you aware of risks (eg, privacy, self-disclosure, time delays)?   - Include informed consent | Include mobile technologies in informed consent; Integrate details of personal and health care mobile technologies use   - Discriminate between types of personal use: significant other/spouse, friends, family; individual/group; personal vs professional - Screen for the patient use of privacy settings for mobile technologies and provide advice based on experience |
| Engagement and interpersonal skills | Discuss impact of mobile technologies use on   - Relationships with others - Professional life - Health care | Ask preferences with mobile technologies and how it/these influenced relationships with family, peers, and professional colleagues   - Positives vs negatives? - Effect on processes of intimacy and emotion   Reflect with patient about the effect on therapeutic relationship   - Communication - Intimacy - Boundaries (see Professionalism)? Compare to other technologies | Provide guidance to patient and family on effective communication using mobile technologies; Instruct on best ways to use mHealth   - An evidence-based app with evidence-based approach - Simplicity with purpose;   Instruct others on impact of asynchronous vs synchronous–and combinations–on communication and the therapeutic relationship; Discuss expectations of parties involved |
| Mental status examination (MSE) | Use mobile technologies to collect information for the MSE | Compare mobile technologies to in-person and telepsychiatric communication; use mobile technologies to check MSE if applicable   - Assess what can and cannot be realistically assessed with mobile technologies | For MSE, use mobile technologies   - Judiciously vs in-person - Adjust administration of tasks (eg, substitute item for impossible task);   Use proxy MSE and physical examination (PE) from another clinician or loved one, if applicable |
| Assessment | Assess if mobile technologies’ use is a relevant issue in personal life and health care; Assess how mobile technologies should be used or *not* be used by a patient and document | Consider the need for collateral info from in-person care or others; Assess healthy/unhealthy use of S mobile technologies in personal life and health care; Integrate mobile technologies components with overall in-person assessments; Demonstrate flexibility and decide with the patient the role of mobile technologies in patient’s needs and preferences | Synthesize information from in-person telepsychiatry, mobile technologies, and other methods (including discordant data); Train, supervise, and consult to optimize assessment, including mobile technologies’ use, problems, need for collateral information; Identify pros/cons of using mobile technologies and for what purpose(s) |
| Management and treatment planning | Integrate mobile technologies into biopsychosocial (BPS) approach (see Decision Support in Knowledge); Consider pros/cons of the decision support tool or app (see Decision Support in Knowledge); Monitor ongoing mobile technologies use as well as documenting memorable and problematic events as they occur; if indicated, focus part of a visit on the use of mobile technologies and other technologies to talk in-depth | Select mobile technology option based on patient preference, skill and need (ie, purpose; see Clinical Decision Support [CDS] in Knowledge); focus on one treatment goal   - App to monitor mood - Capture day-to-day accurate accounts of a patient’s emotions, functioning, and activity (ie, ecological momentary assessment [EMA]);   Blend mobile technologies with regular clinical discussions, facilitate reflection and assess effect on the therapeutic relationship–in and between sessions; Identify safety/risk factors of mobile technologies use (eg, giving advice on medication); back-up plan for failure; Triage complex, urgent/emergent issues to synchronous (telephone and in-person) care; Weigh pros/cons of mobile technologies use vs other technologies and discuss options for informed consent; document | Use BPS outline with prioritization, with adjustments for technology; Select “best” mode for a given task: mobile technologies, email/text, telephone and in-person; Be aware of legal, billing, and jurisdictional issues for medication; Research and disseminate procedures to prevent problems and manage clinical and administrative issues; Advise on specific behavioral health problems and specific patient populations with relative/absolute contraindications |
| Clinical decision support (CDS) | Use mobile technologies within for decision-making and care; Review examples with learner/supervisor | Adjust mobile technologies within parameter(s) to for decision-making; Help patients, learners and staff use decision support tools based on evidence; Prioritize mobile technologies options, email and tools that integrate into the EHR | Instruct on how to use pre and intraplatform data feeds (eg, questionnaire upload) into EHR to improve quality of care and be efficient |
| Patient and family education | Understand reliable/healthy and unreliable/unhealthy mobile technologies options; Value of using mobile technologies in health care and when to use it | Recommend how to use mobile technologies in health care (eg, tips on how, when and what uses are appropriate; what should be posted and what should not); Offer “good” choices for specific purpose(s) for mobile technologies use in personal life and health care | Instruct with examples, principles and evidence for responsible mobile technologies use by people, patients, and organizations (eg, schools); Provide firsthand knowledge of the pros/cons of different mobile technologies for health care |
| Administration and documentation | Adhere to clinic, health system and professional requirements for in-person care and consider amendments for mobile technologies and other technologies   - Document in informed consent - Document key events;   Seek supervision/advice for nonroutine events, if needed | Develop standard language for consent form, treatment plan and sentinel events on the pros/cons of mobile technologies use; Adapt current practices and develop new policies/procedures for Mobile technologies and other technologies; Seek advice in advance to plan; document; Consider/attend to business and financial issues (eg, pros/cons of time used) | Instruct on in-person, telepsychiatry and mobile technologies applications related to documentation, privacy, and billing |
| Medicolegal issues: privacy, confidentiality, safety, data protection/int-egrity and security | Identify and adhere to laws and regulations in the jurisdiction(s) of practice and of that of the patient; Clarify if mobile technologies access is public, private and within electronic health record (EHR); Advise patients to communicate and send data privately (eg, secure email within EHR not Gmail) | Apply in-person relevant laws and regulations in any/all jurisdiction(s) to mobile technologies, and if necessary, adjust clinical care; Educate patient about mobile technologies and adapt existing laws if none exist for it/these and other telepractice; Obtain clinical and legal advice, as applicable | Teach/consult on in-person laws and regulations for mobile technologies and other technologies; Develop strategies to adapt legal and regulatory principles from in-person to care mobile technologies use; Update/consult with regulatory boards, health authorities and professional organizations |
| **Interpersonal and Communication Skills** | | | |
| Communication | Be flexible in discussing mobile technologies use and communication; Discuss problems if they arise with asynchronous options and arrange alternative options; Seek advice on merit and method of responses, if any, to patient’s communication | Discuss scope of communication with mobile technologies, clarify expectations and anticipate problems (eg, feasibility of checking mobile technology at other sites, clinics); Discuss scope, timing and agreed upon plan(s) for asynchronous options; Make brief, clear mobile technologies communications to acknowledge, clarify and triage to in-person care | Identify and trouble-shoot communication issues related to technology and other; Educate and provide consultation to colleagues about asynchronous technology use; Clarify expectations and potential ambiguous (ie, multiple) meanings of acronyms, abbreviations, and such communication |
| Evaluation and feedback | Periodically evaluate examples of decision-making and care with patient/supervisor | Evaluate mobile technologies use, adjust regular evaluation parameter(s), and incorporate real-time examples in ongoing fashion with patient/learner/supervisor | Teach, consult and role model feedback skills related to synchronous and asynchronous (ie, mHealth) technologies; Develop teaching cases/in situ examples |
| Cultural, diversity, and social determinants of health; attend to language issues | Consider culture and diversity issues, related to mobile technologies and other technologies   - How social determinants affect synchronous and asynchronous health care - Access to mobile technologies - Sentinel events | Ask patient if/how culture impacts use and preferences for mobile technologies and other technologies; Promote reflection and awareness of how social determinants and mobile technologies intersect; Observe, adjust, and manage language and communication issues (eg, emoticon use) | Include mobile technologies use in cultural formulation interview, if applicable; Instruct on generalizations (and how to avoid stereotypes) of how culture may affect mobile technologies use and impact treatment/patient care; Consider consultation |
| Special populations | Notices positive and negative trends in patient populations (eg, generation Y or Z, autism spectrum) | Consider preferences of mobile technologies use (eg, adolescent, Veteran with posttraumatic stress disorder); Be aware of trends across asynchronous technologies (eg, email/text, apps) | Instruct on how to adapt assessment and management approaches according to differences |
| **Professionalism** | | | |
| Attitude | Show interest about patient’s use of mobile technologies; Demonstrate capacity for self and others’ reflection | Express interest, be nonjudgmental, and be spontaneous in discussing technology; Engage via mobile technologies within appropriate expectations, purpose(s) and safeguards in place | Provide leadership to colleagues on organizational policy or curricula for mobile technologies and professionalism |
| Integrity and ethical behavior | Maintain integrity by adhering to professional and governmental guidelines; Recognize boundary, privacy, and confidentiality issues with mobile technologies communication | Uses clinical judgment and ethical principles to purposely use mobile technologies to collect and transfer patient information; Reflect on personal vs professional contexts and potential micro- and macro-boundary violations (eg, texting patient after clinical hours as “convenient”); Recognize that personal information (eg, health) may be accessible and monitor | Role model, teach/consult others to manage complicated ethical issues related to the use of mobile technologies in clinical practice and related to professional identity; Research and develop approaches uphold quality of the therapeutic relationship and communication for care |
| Scope and therapeutic objective(s) | Practice within scope(s) and discuss expectations with patient; Keep focus on shared primary objective of care | Attend to and evaluate how mobile technologies may alter in-person scope issues; Trouble-shoot problems; Assess if mobile technologies licensed and reputable; avoid fraudulent practices; and market within regulations (eg, Federal Trade Commission substantiation rule) | Develop and teach/consult use on mobile technologies to adjust for patient populations (eg, age, illness/disorder); Evaluate and advise on complex cases (eg, high-risk populations, legal complications) |
| **Systems-based practice** | | | |
| Interprofessional education (IPE) and team work | Learn about mobile technologies and other technologies and share information with others | Discuss/teach mobile technologies issues with team members to enhance care; Weigh pros/cons of mobile technologies related to communication, privacy, and clinical productivity | Assess technology info from IPE team point-of-view in systems; Adjust assignments/roles; Role model/give feedback |
| Safety (see Patient Care and Professionalism) | Educate patient to call and set up additional appointment for emergencies; Seek advice/consultation, when needed | Prevent, identify and risk stratify potential problems based on past history in case mobile technologies; Educate patient to use in-person or synchronous (eg, video, telephone) communication for emergencies | Adjust risk and its management to mobile technologies, based on in-person and technology-based system practice; Instruct others in pitfalls of mobile technologies use in health care |
| Models, practices, and systems of care | Aware of consultation, evaluation, triage, and management clinician roles; Practice with principles of evidence-, measurement- and population-based care; Incorporate workflow between technology devices (eg, desktop, portable) into EHR | Give input to administration on (in)efficiencies and opportunities to integrate mobile technologies data in workflow/EHR for decision-making; Distinguish between principles of evidence-, measurement- and population-based care related to mobile technologies; Choose when to use mobile technologies vs email/text and other technologies when mobile across sites; Apply sensors, remote monitors, and other devices (eg, home) in accordance to medicolegal scope and standards | Engage providers and consultants about the role of mobile technologies; Consider what part, if any, of the “therapeutic hour” is used for mobile technologies, email/text and other technologies; Instruct on home health options related to mobile technologies to enhance clinical evaluation and treatment |
| **Practice-based Learning** | | | |
| Evaluation approach | Learn from/participate in global evaluations from patients, interdisciplinary team, and clinic/hospital about in-person and technology-based care related to mobile technologies | Be aware that in-person, mobile technologies, and other technology-based care have similarities and differences; suggest improvements; Develop/promote attitudes and skills for consistency, quality/specificity, and stability of evaluation | Teach/consult on practice standards of evaluation and adjustments for mobile technologies; Compare/contrast information across professions, disciplines, fields, states/provinces/countries; Shift policies and procedures |
| Quality improvement (QI) | Participate in chart review, case/M&M conference and other activities related to in-person and technology-based care | Apply/adapt in-person QI principles to mobile technologies to adjust assessment and care; Educate participants on technology-specific principles and measures | Develop QI strategies to adhere to and adapt legal, regulatory, and ethical standards (eg, privacy, access); Teach/consult on how to analyze, select and evaluate QI options |
| Learning, feedback, and teaching practices | Add technology-based learning opportunities to regular activities; Consider role of technology in care | Continue lifelong learning via seminars, cases, and system discussions; Seek out technology-specific education; Develop additional technology-specific education short- and long-term; Assess effect(s) of technologies on care | Research learning and teaching methods to streamline educational approach and evaluation; Determine best context(s) for teaching and learning mobile technologies (eg, supervision, seminar/cases) |
| **Knowledge** | | | |
| Definition of mobile technologies | Recall definition of mobile technologies; Name 2 or 3 mobile technologies with pros/cons | Describe mobile technologies definitions and various uses, purposes, and risks/benefits to patients; Professionally familiar with 2-3 mobile technologies and a mHealth platform for professional use; Serve as resource for others | Teach multiple mobile technology varieties; consults with colleagues; Instruct on the approach to mHealth, selections of apps and pros/cons |
| Evidence-base | Know basic “do’s or don’ts” of mobile technologies for clinical care, as adapted from in-person care | Knows the data, concepts, and principles of mobile technologies use (eg, standards, guidelines)   - How to assess if an app is evidence-based - How to use an app in an evidence-based clinical approach | Teach/consult to health care colleagues locally and nationally to develop best practice guidelines using the clinical evidence on mobile technologies |
| Problem-solving and prevention | Recognize and report problems; Perform basic how mobile technologies are part of assessment and treatment; Explain ways in which a patient can better learn how to use a mobile technologies product | Evaluate new products/options and the pros/cons; Assess performance issues of current systems or products; Assess user requirements and determine best match for patients and other participants with technology options; Diagnose complex problems and resolve nonroutine problems that affect team; Serve as a resource to others; Know how to request technical assistance | Research and disseminate personal vs professional aspects of mobile technologies platforms; Keep up to date with latest mobile technologies developments (eg, privacy) |
| Patient care | Ability to answer questions, discuss and adjust mobile technologies in comparison to in-person care, including consent, privacy, data protection/integrity and security safety and documentation; Aware of mobile technologies security measures (ie, password protection) | Answer questions/teach, discuss/clarify and adjust/develop options for mobile technologies in comparison to in-person care in additional areas of scope of practice, communication, culture, and diversity, ethics, and care models; Aware of mobile technologies security measures (ie, password protection bypassed if incoming call “goes around” security measure) | Demonstrate extensive knowledge of mobile technologies to advise colleagues on practical knowledge of how to mitigate them |
| Decision support | Understand the role of technology in initiating, enhancing, and monitoring decision making; Use mobile technologies within for decision-making and care; Review examples with learner/supervisor | Find and evaluate the role of mobile technologies in initiating, enhancing, and monitoring decision making; Compares the pros/cons of mobile technologies in to manual operations (eg, error identification, duplicative processes) for an acute vs chronic condition; Help patients, learners and staff use decision support tools based on pros/cons; Realize that adjustments to mobile technologies parameter(s) are possible to aid in decision-making | Demonstrate extensive knowledge of mobile technologies to advise colleagues on how to evaluate decision support tools; Instruct on fundamentals of adjusting to mobile technologies parameter(s) to aid in decision-making |
| Risks of using mobile technologies | Identify 1 potential patient risk of mobile technologies use (ie, privacy violation); Identify 1 potential provider risk of mobile technologies use (ie, boundary or privacy violation) | Identify 2-3 potential patient risks of mobile technologies use and advises how to prevent, mitigate, or eliminate them (eg, use privacy settings; avoid self-disclosure; manage cyber-bullying); Identify 2-3 potential provider risks of mobile technologies use and prevent, mitigate, or eliminate them (eg, use privacy settings) | Demonstrate extensive knowledge of mobile technologies risks and advises colleagues; Anticipate common pitfalls and how to prevent/mitigate them |
| **Technology** | | | |
| Adapt to technology | Use basic etiquette; Identify differences between care in-person, telepsychiatry and mobile technologies; Clarify/spell out brief communications; Keep mobile technologies contact proportioned and specific to goals of the treatment plan | Clarify expectations in-person rather than asynchronously; Acknowledge and engage the patient, as clinically indicated (eg, depressed patient ability to use an app); Expect and plan for differences between participants; Prevent, identify, and manage barriers, obstacles, and miscommunications; Adjust how to “project” self and express empathy | Generally, avoid humor, self-deprecatory remarks, and jokes via synchronous methods; Analyze what actually happened and make adjustments for next time; Show meaningful ways to express empathy |
| Technology operation | Pilot 1 or 2 mobile technologies with peers to learn communication options | Gain experience with mobile technologies; Navigate options, if needed, and advise patients relative to goal and purpose | Research and teach/consult on best approaches for mobile technologies operations for clinical quality (eg, hard/software; accessories; and common trouble-shooting strategies) |

^a^ Technically mHealth options may be synchronous, although most are asynchronous, so that term is used.
