# Supplementary material for: A Framework for Competencies for the Use of Mobile Technologies in Psychiatry and Medicine: Scoping Review
Source: JMIR Mhealth Uhealth. 2020 Feb 21;8(2):e12229. doi: 10.2196/12229 (PMC7060500; doi:10.2196/12229)
Supplement: Multimedia Appendix 2 [file mhealth_v8i2e12229_app2.docx]

Multimedia Appendix. Teaching, assessment, and evaluation methods for mobile technologies (mobile Health, smartphone, and apps) clinical competencies.

| Teaching and/or learning method | Context(s) | Competency domain(s) addressed | Learner assessment methods |
| --- | --- | --- | --- |
| **Didactic teaching** | | |  |
| All methods | Dependent on venue/setting | Knowledge, Patient Care, Systems-Based Practice, Technology—primarily knowledge at the precompetency and competency levels;  Provide content knowledge, but less effective for developing attitudes and skills | Written tests: multiple-choice and short-answer questions;  Audience participation system |
| Brief didactic | Clinical setting with mobile technologies applied to care (eg, replace nonsecure technology with one for privacy) | Focus: solve immediate question/dilemma (eg, emergency, privacy);  Focus: engage/help learners contextualize day-to-day events and gain further education;  Focus: provide research, trends, and relevance of mobile technologies; compare to email/text and synchronous modes (eg, in-person, telepsychiatry or telephone) | Application to context (eg, pre- and posttest);  Written tests: multiple-choice and short-answer questions |
| Grand rounds or longer didactic | Classroom in person, by video, or webinar |  |  |
| **Case-based Learning** | | |  |
| Brief vignettes | Individual, pair/share and problem- and/or team-based learning about mobile technologies issue for patients with primary in-person, telepsychiatry or other technology-based care (eg, patient reports suicidal ideation (SI) in filling out Patient Health Questionnaire-9 (PHQ-9); how to triage this) | Patient Care, System-Based Practice, Technology – knowledge for all levels of competency;  Provide content knowledge and effective for developing attitudes and skills;  Focus: deepen content knowledge and apply/generalize knowledge to real-life examples;  Focus: good for complex clinical situations to develop steps of treatment/management plans (eg, emergency);  Focus: effective for highlighting key asynchronous events that are random and ill-timed between clinic visits and supervisory hours | Case-based written tests: multiple-choice, pre- and posttests and/or short-answer questions (eg, next best step is…);  Oral presentation with preassigned case (such as flipped classroom) or in session case |
| Complex, multi-step cases |  |  |  |
| **Clinical Care with Patients (**See Patient Care in Table 1**)** | | |  |
| Observing faculty | Live patient interview in-person or telepsychiatry (eg, initial evaluation) in which/mobile technologies issue arises, is screened for and/or the focus of the conversation | Patient Care, Communication, Systems-Based Practice, Technology – primarily at the precompetency level;  Adjusts attitudes and may be used to demonstrate complex skills by faculty role modeling;  Foci: develop interviewing skills about realistic expectations, uni- versus bidirectional communication and data transfer;  Focus: purposeful vs random use of mobile technologies, outcomes, and monitoring;  Focus: inappropriate use (eg, emergency; after hours; SI and homicidal ideation (HI) comments; sending an app on how to be a ‘better’ anorexic);  Focus: educate patient about common errors/pitfalls of mobile technologies); Focus: create a culture to openly discuss key issues about mobile technologies | Evaluation by observation supplemented by review of mobile technologies and chart;  Research/analysis on trends with mobile technologies;  Develop and disseminate policies related to similarities/differences of technologies to in-person care |
| Group observed or co-interviewing | Group interview room (in-person or telepsychiatry): learners take-turns with assessment; group and supervisor feedback; Can also use separate room or 1-way mirror | Patient Care, Communication, Professionalism, Technology – precompetency and competency levels;  Systems-Based Practice (eg, decision support) – primarily precompetency level;  Focus: develop interviewing skills and apply knowledge to screen, evaluate and plan mobile technologies options;  Focus: employ group/discussion and reflection to explicitly explore elements of scope of practice, professionalism, and cultural and social factors;  Focus: build consensus on pros and cons of mobile technologies; share experiences | Mini-CEX (Clinical Evaluation Exercise) completed by faculty on each learner and direct verbal feedback;  Peer-recorded written evaluation; peer review |
| **Professional Reflection, Monitoring and Hygiene related to Patient Care and Supervision** | | |  |
| Caseload-based self-reflection, presentation, discussion, and decision making | Reviews own personal and professional use of mobile technologies to identify pros/cons   - Similarities/differences - How projecting image - ‘Bad’ outcomes;   Supervision: share findings in individual and/or therapy supervision   - Purpose(s), outcomes, and adjustments of mobile technologies use - Patient errors or those ill-equipped to use technology;   Review unexpected events between live visits | Patient Care, Communication, Systems-Based Practice, Professionalism, Technology – all levels of competency;  Effective for developing attitudes and skills, but less so for gaining knowledge;  Focus: synthesis in a complex case or pattern analysis across a population;  Focus: evaluate reactions and meaning of events, including transference and countertransference;  Focus: develop and adjust management plans (eg, triaging and next steps; preserving privacy and boundaries);  Focus: identify policy factors and knowledge gaps;  Focus: assess systems- and population-level thinking, decision support, workflow, and resource allocation;  Examples:  Correct inaccuracies, prevent/manage boundary violations (eg, personal picture or video transferred; patient ‘friends’ trainee on social media);  Patient adherence to expectations (eg, weekly app completion);  Good to assess events and review decision-making on correspondence | Oral presentation in supervision and/or group, with follow-up formal didactic one (eg, grand rounds);  Chart review of patient treatment plan for decisions;  Policy development/review/adapt to mobile technologies for clinic;  Feedback through peer review process;  Workflow illustration for decision support;  Follow-up report on interventions and impact;  Longitudinal, cumulative evolution of clinical skills and relationship |
| In time supervision in-person or at distance on critical incident (eg, emergency) | Untoward expected and/or unexpected event(s) (eg, SI, HI, bullying and/or aggressive posturing/threat, sexual overtone using mobile technologies) | Patient Care, Systems-Based Practice, Professionalism – primarily precompetency to competency levels;  Provides content knowledge to manage situation and skills more so than attitudes;  Focus: immediate adjustment of management plans (eg, triage patient to live visit, if necessary, for dangerousness assessment);  Focus: engage emergency response systems including authorities for duty to warn;  Focus: engage/help learners contextualize day-to-day events and gain further education;  Focus: evaluate meaning of events (eg, transference), adjust treatment plans and pre-emptive planning (eg, preventing, triaging events) | Timeliness of request for supervision;  Initial assessment of potentially emergent situation and plan of action;  Feedback in real time;  Follow-up report on interventions and impact;  Chart review (eg, documentation related to specific language of mobile technologies, triage, and response |
| Simulation on decision support – with video or standardized patients | Use of cases, standardized patients, or video clips   - Find, research, and select decision support tool(s) based on dataset - Adjust decision based on outcomes and/or additional dataset | Patient Care, Knowledge, Systems-Based Practice, Practice-Based Learning – all competency levels;  Provides balance of content knowledge to manage situation, skills acquisition, and attitude development/adjustment;  Focus: ability to perform, analyze, receive feedback, and reflect on own performance and style;  Focus: ideal for more advanced skills that require start-stop and in-action reflection and feedback (eg, administering tools; challenges with safety/risk; rapidly changing data input) | Feedback in real time;  Equivalent to a technology objective structured clinical examination (OSCE) |
| **Quality Improvement (QI), Evaluation and Research** | | |  |
| Case write-ups | Trainee and mentor submission for committee, conference presentation and/or final report/publication;  Evidence-, measurement- and population-based care; systems-level thinking and health planning/resource allocation | Systems-Based Practice, Practice-Based Learning – all levels of competency;  Effective for developing attitudes and skills, but less so for gaining knowledge;  Focus: synthesis of a case short- and long-term and application to other cases;  Focus: good introduction to administration, evaluation, and policy-oriented factors (eg, advanced knowledge gaps);  Focus: evaluate apps to see if they are outcome- and evidence-based; develop an approach to use them in an evidence-based fashion | Evaluation of literature search;  Evaluation of written synopsis;  Verbal presentation, discussion, and group feedback;  Peer, interprofessional, supervisor and administrator feedback |
| Literature reviews |  |  |  |
| QI presentation/project with interprofessional team, informatics team and administration; QI-related (eg, length of stay, informatics) committee |  |  |  |
| **Role as Educator** | | |  |
| Provide didactic sessions | Learner participates/leads discussions on mobile technologies | Knowledge, Technology, Systems-Based Practice, Practice-Based Learning – all competency levels;  Develop attitudes and skills more so than content knowledge;  Focus: learn to work with an interprofessional team; Focus: adapt communication to multiple people;  Focus: develop advanced skills, such as enhancing capacity and competencies in distance staff (eg, teaching to use technology, decision support tools and integrate data throughout system);  Focus: evaluate apps to see if they are evidence-based and develop and approach to use them accordingly | Reflection journal for observation;  Questions by participants;  Evaluation forms completed by participants;  Feedback solicited from participants of interest (eg, content expert, training director, informatics director) |
| Group and interprofessional learning (eg, journal club) | In-person, video and/or Web-based | Systems-Based Practice, Practice-Based Learning – all competency levels;  Focus: develop attitudes and skills more so than content knowledge;  Focus: enhance interprofessional and collaborative skills;  Focus: build professionalism skills;  Focus: dissemination, establish community/culture of practice and outreach across institutions | Evaluation forms completed by participants |
